# The Effectiveness of Biostimulation, Bioaugmentation and Sorption-Biological Treatment of Soil Contaminated with Petroleum Products in the Russian Subarctic

**DOI:** 10.3390/microorganisms9081722

**Published:** 2021-08-13

**Authors:** Vladimir A. Myazin, Maria V. Korneykova, Alexandra A. Chaporgina, Nadezhda V. Fokina, Galina K. Vasilyeva

**Affiliations:** 1 Institute of North Industrial Ecology Problems—Subdivision of the Federal Research Centre “Kola Science Centre of Russian Academy of Science”, 184209 Apatity, Russia; sashka-26.11.91@mail.ru (A.A.C.); nadezdavf@yandex.ru (N.V.F.); 2 Agrarian Technological Institute, Рeoples’ Friendship University of Russia (RUDN University), 117198 Moscow, Russia; 3 Institute of Physicochemical and Biological Problems in Soil Science Russian Academy of Science, 142290 Puschino, Russia; gkvasilyeva@rambler.ru

**Keywords:** petroleum-contaminated soil, bioremediation, sorbents, microfungi, mycoremediation, hydrocarbon-oxidizing microorganisms, dehydrogenase activity, granular activated carbon, peat

## Abstract

The effectiveness of different bioremediation methods (biostimulation, bioaugmentation, the sorption-biological method) for the restoration of soil contaminated with petroleum products in the Russian Subarctic has been studied. The object of the study includes soil contaminated for 20 years with petroleum products. By laboratory experiment, we established five types of microfungi that most intensively decompose petroleum hydrocarbons: *Penicillium canescens* st. 1, *Penicillium simplicissimum* st. 1, *Penicillum commune*, *Penicillium ochrochloron*, and *Penicillium restrictum*. One day after the start of the experiment, 6 to 18% of the hydrocarbons decomposed: at 3 days, this was 16 to 49%; at 7 days, 40 to 73%; and at 10 days, 71 to 87%. *Penicillium commune* exhibited the greatest degrading activity throughout the experiment. For soils of light granulometric composition with a low content of organic matter, a more effective method of bioremediation is sorption-biological treatment using peat or granulated activated carbon: the content of hydrocarbons decreased by an average of 65%, which is 2.5 times more effective than without treatment. The sorbent not only binds hydrocarbons and their toxic metabolites but is also a carrier for hydrocarbon-oxidizing microorganisms and prevents nutrient leaching from the soil. High efficiency was noted due to the biostimulation of the native hydrocarbon-oxidizing microfungi and bacteria by mineral fertilizers and liming. An increase in the number of microfungi, bacteria and dehydrogenase activity indicate the presence of a certain microbial potential of the soil and the ability of the hydrocarbons to produce biochemical oxidation. The use of the considered methods of bioremediation will improve the ecological state of the contaminated area and further the gradual restoration of biodiversity.

## 1. Introduction

Currently, because of anthropogenic activities, there is large-scale pollution of the environment with toxic substances. Petroleum and petroleum products are major pollutants among all contaminants entering the environment. Contamination with this type of pollutant is also relevant for regions where petroleum production and refining are not carried out. In this case, petroleum depots, objects of the fuel and energy complex, and large industrial enterprises with motor transport divisions and gas stations in their structure become potentially dangerous sources of soil pollution. Spills of petroleum or petroleum products and the pollution of natural environments occur during their storage, transportation, shipment and bunkering, as well as resulting from the wearing down of equipment.

The soil undergoes a special load—the degradation of its morphological and physicochemical properties, significant suppression of the soil’s microorganism activity and a decrease in its self-cleaning ability [1,2]. Contaminated soil ceases to perform the main ecosystem functions associated with its chemical, physicochemical, and biochemical properties. As a result of the degradation processes, the content and reserves of humus in the soil decrease; its qualitative compositional changes; the processes of nitrogen fixation, ammonification, nitrification, and mineralization are inhibited; the nutrient regime of the soil deteriorates; its enzymatic activity decreases; and the pH value and redox potential of the soil often change [2,3].

The need to develop effective methods of treatments and technologies for recovering petroleum-contaminated soils is one of the main environmental problems in the northern regions of Russia. In the Arctic zone of Russia, there is currently a problem with eliminating the consequences of accumulated environmental damage. There are areas contaminated with fuels and lubricants as a result of past economic activities where soil cleaning and recovery have not been carried out for many years [4,5].

Microbiological methods of environmental treatment from petroleum pollution are ecologically promising. The currently applied methods of cleaning oil-contaminated areas provide for the excavation of contaminated soil, its movement, and cleaning by physical, chemical or biological methods. Cleaned soils are used mainly in road construction. To restore the fertile layer in the cleaning area, natural soil is used. Remediation requires significant excavation and storage of soil, which will lead to an increase in the disturbed land area. All these manipulations change the natural soil profile, generate waste, and use additional soil resources [6]. These include biostimulation, which is based on the addition of extra sources of carbon and nutrients to stimulate microbial activity, and bioaugmentation—the introduction of cultures of petroleum-oxidizing microorganisms [3,7,8,9]. Recently, special attention has been paid to the possibility of using fungi for bioremediation (mycoremediation). Fungi are able to survive in adverse environmental conditions as well as to transform pollutants that are inaccessible or hardly accessible to soil bacteria [10,11,12,13]. There are a number of works demonstrating the successful application of mycoremediation [14,15,16,17,18]. The correct selection of fungi with a powerful degrading potential is the main criterion for the successful application of this technology.

The use of the bioremediation method does not always give good results, especially in arctic and subarctic conditions. This is due to the low biological activity of the soils of the northern regions of Russia and the high vulnerability of the natural ecosystems. The solution to this problem could be the technology of sorption-biological soil treatment based on the use of various sorbents [19,20,21]. The use of sorbents accelerates the processes of bioremediation and land reclamation, thereby expanding the possibilities of biological treatment. Sorbents serve as a source of nitrogen and phosphorus and a place of localization of microorganisms-destructors, and they increase the moisture capacity and aeration of soils [22]. Studies carried out earlier in the central region of Russia made it possible to create an effective method of the sorption-biological treatment of soils contaminated with organic pollutants [23,24,25,26,27]. The use of granular activated carbon (GAC) can accelerate the processes of the bioremediation of soils contaminated with diesel fuel, used engine oil, and petroleum [28,29].

In laboratory conditions, the efficiency of the sorption-biological treatment of the contaminated soils of the Murmansk region has been shown. The introduction of optimal doses of sorbents into the soil make favorable conditions for the activation of indigenous or specially introduced microorganism-degraders [30].

The aim of this work is to determine the influence of bioremediation methods (biostimulation, bioaugmentation, sorption-biological treatment) on biological indicators and the content of hydrocarbons in contaminated soil.

## 2. Materials and Methods

### 2.1. The Territory of the Study

The study was carried out in the area of Mount Kaskama, located in the north of the European part of Russia (Murmansk region (69°16′16.1″ N; 29°27′37.9″ E). This site belongs to the boreal–subarctic forest–tundra zone with a predominance of sparse pine forests (*Pinus sylvestris lapponiсa*) [31].

### 2.2. Climate

This territory belongs to the Atlantic–Arctic region, which has two climatic zones: subarctic and temperate. The strong influence of the Atlantic has a softening effect on air temperature and increases humidity. The average air temperature in the coldest months (i.e., January, February) is −11 °C and in the warmest month (July) is + 12 °C. The average wind speed is 3.7 m/s and relative humidity is 68% [32]. The polar night is typical in winter and the polar day in summer.

### 2.3. Soils

**Figure 1:**
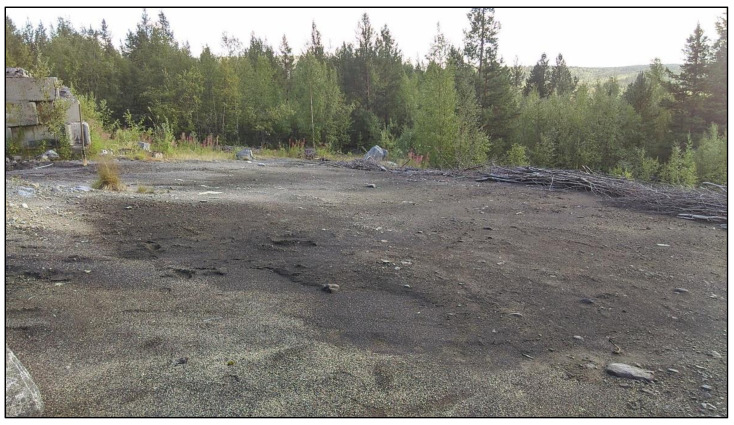
Contaminated area.

**Figure 2:**
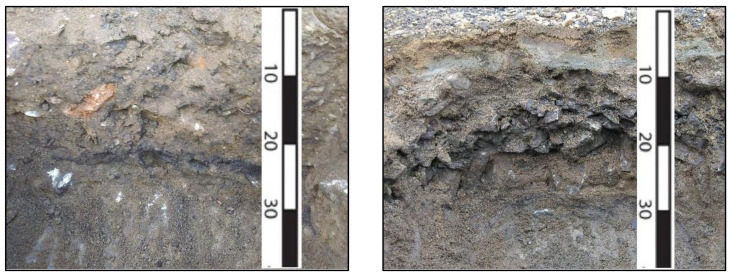
Soil profile in the contaminated area.

The undisturbed areas are dominated by folic albic podzol with slightly humified forest litter, a pH of 4.5–5.1, and a mineral profile of up to 40–60 cm thick [33,34]. As a result of anthropogenic impact over a long time, a contaminated site about 0.1 hectares has formed. The surface of the contaminated site is devoid of an organogenic horizon (Figure 1) and is represented by a sandy fraction with a large amount of stony inclusions (Figure 2). The density of the soil is 1300 kg/m^3^, and the content of organic carbon in the 0–7 cm layer is 4.47–5.43%.

### 2.4. Sorbents

As sorbents, we used GAC of the GAU VSK brand and neutralized peat. The GAC with a granule size of 2–3 mm consists of 87–97% carbon, and the rest is represented by oxygen- and sulfur-containing high-molecular compounds, metals, etc. The specific surface area of activated carbon is 550–2400 m^2^/g, and the sorption capacity in relation to hydrocarbons is 200–980 g/kg [35].

The organic sorbent is milled peat with a low degree of decomposition (no more than 35%), the mass fraction of moisture is no more than 65%, the pH_KCl_ is no less than 5.2, and the organic matter content is no less than 70%.

### 2.5. Associations of Hydrocarbon Oxidizing Microorganisms

In the field experiment, we used associations of hydrocarbon-oxidizing microorganisms (HOM), consisting of microfungi and bacteria that destroy petroleum products, isolated earlier from the contaminated soils of the Kola Peninsula.

Previously, we investigated the petroleum-degrading activity of 81 fungi strains from 30 genera (Acremonium, Alternaria, Aspergillus, Aureobasidium, Botrytis, Cephalosporium, Cephalotrichum, Chaetomium, Сladosporium, Clonostachys, Fusicolla, Gibberella, Gibellulopsis, Gongronella, Humicola, Lecanicillium, Mucor, Penicillium, Pseudogymnoascus, Rhizopus, Rhodotorula, Scopulariopsis, Stachybotrys, Streptothrix, Talaromyces, Torula, Trichoderma, Ulocladium, Umbelopsis, Wallrothiella) in laboratory experiments. The cultivation of fungi was carried out in Erlenmeyer flasks with 50 mL of Czapek’s liquid nutrient medium of the following composition in g/L: NaNO_3_ at 3.0, KH_2_PO_4_ at 1.0, MgSO_4_ at 0.5, KCl at 0.5, and FeSO_4_ at 0.01. We inoculated 50 mL of the nutrient medium with 5 mL of a microfungi suspension with a density of 10^5^–10^6^ CFU in 1 mL. As a carbon source, oil from Prirazlomnoye field (Arctic Oil (ARCO) is distinguished by high density (about 910 kg/m^3^), medium sulfur content and low paraffin content) was introduced into the medium at a concentration of 1% by volume. The experiment was repeated 3 times. A sterile petroleum-free medium without fungi was used as a control. Incubation was carried out in a thermostat at 27 °C for 14 days. The biomass of microfungi was determined by drying it at 105 °C to absolutely dry weight.

The petroleum degradation by the most active strains of microscopic fungi isolated of the previous experiment (i.e., *Penicillium canescens* st.1, *Penicillium commune, Penicillium ochrochloron, Penicillium restrictum, Penicillium* *simplicissimum* st.1) was studied in a time gradient: after 1, 3, 7, and 10 days.

At the final stage, the degrading activity of fungi associations and bacteria was investigated. Active strains of fungi (*P. commune, P. simplicissimum* st. 1, *Penicillium canescens* st. 1) and hydrocarbon-oxidizing bacteria (*Microbacterium paraoxydans, Pseudomonas fluorescens, Pseudomonas baetica, Pseudomonas putida*), previously isolated from the soils of the Kola Peninsula. were used in laboratory experiment [36,37]. Bacterial biomass was produced in meat-peptone broth in a laboratory fermenter (BIOSTAT^®^ A plus, Sartorius, Germany) at a temperature 27 °C and aeration for 3 days. The density of the bacterial suspension was 10^8^–10^9^ cells/mL. Cell density was determined by fluorescence microscopy using polycarbonate membrane filters. Regions of the 16S and Internal Transcribed Spacer (ITS) gene obtained from these bacterial strains were deposited in the NCBI GenBank database under the accession numbers KM 288708, KM 288709, and KM 216318. Microscopic fungi were produced in the same way as in the previous experiment. To prepare an association of hydrocarbon-oxidizing microorganisms, bacterial and fungal suspensions were mixed in equal proportions in meat-peptone broth.

### 2.6. Experiment Design

Experimental plots with a size of 1 m^2^ in triplicate were laid at the western foot of the mountain. The design of the field experiment is presented in Table 1.

To activate aboriginal petroleum-degrading microorganisms (biostimulation) and remove nutritional deficiencies, complex mineral fertilizer (16% N, 16% P_2_O_5_, and 16% K_2_O) and dolomite flour (CaCO_3_ content of 80%) were added to all plots except for the control (K). The amount of fertilizer was calculated based on the content of hydrocarbons in the soil (ratio C:N = 100:1), and the amount of dolomite flour was calculated based on the pH value. After the addition of fertilizer and dolomite flour, an association of HOM (BP) in the amount of 2 L/m^2^, GAC in the amount of 1 and 3 wt. % (GAC1 and GAC3, respectively), and peat (Peat) in an amount of 16 l/m^2^ were added to various sites. The one variant (NPK) with fertilizer and dolomite flour was left without adding sorbents and HOM. The top layer of the soil in all variants was mixed to a depth of 7–10 cm. After 12 months (at the beginning of the next growing season), the soil amendments were repeated. Soil samples were taken from each experimental site from a layer of 0–7 cm. The experiment was started in June 2019, the observation period was 15 months. Samples were taken during the growing season (0 and 12 months) and at the end of the growing season (3 and 15 months).

### 2.7. Determination of the Total Petroleum Hydrocarbons Content in Soil

The content of total petroleum hydrocarbons (TPH) in the soil was determined by infrared spectrometry by using the AN-2 analyzer [38]. This method is based on the extraction of TPH from the soil with tetrachlorocarbon, the separation of oil products from the polar hydrocarbons in the column filled with aluminum oxide, and the further spectrophotometric identification of hydrocarbon content according to the absorption intensity of infrared radiation at fixed wavelengths 540 nm (Analyzer of oil products AN-2 “Neftekhimavtomatika”).

### 2.8. Determination of Physical-Chemical Properties of Soil

The actual acidity of the soil was determined in water soil extracts in a ratio of 1:5 by the potentiometric method on a laboratory instrument, a Radelkis OP-300 pH meter with a combined pH electrode [39]. The instrument was calibrated in the pH range from 4.01 to 6.86 using buffer solutions prepared from standard titers.

Soil humidity was measured by drying soil samples at 105 °C for at least 6 h to constant weight. Soil temperature was determined in the field at a depth of 5 cm using a Hanna HI 145 electronic thermometer.

### 2.9. Biological Parameters

For microbiological analysis on each experimental plot, soil samples were taken from a layer of 0–10 cm. The number of cultivated microscopic fungi was determined by the submerged inoculation method on a nutrient medium with the addition of lactic acid (4 mL/L) for bacteriostatic action. The number of cultivated bacteria was determined by the surface inoculation method on a nutrient medium for hydrocarbon-oxidizing bacteria with the addition of petroleum. 

The activity of soil dehydrogenase was determined by the colorimetric method based on the reduction of the colorless salt of 2,3,5-triphenyltetrazolium chloride to red triphenylformazan [40,41].

### 2.10. Statistical Processing

Statistical analysis of the data was carried out using the applied programs Statistica 6.0 and Microsoft Excel 2007. Student's t-test was used to determine the reliability of the coefficients. Pearson's method (significance level 0.05) was used to calculate the correlation coefficient (r).

## 3. Results

### 3.1. Degradation Activity of Microfungi in Laboratory Experiments

Based on the results obtained, a scale of degrading activity of microfungi to petroleum hydrocarbons has been developed (Table 2). All species were categorized into three groups:

(a) Species with high hydrocarbon oxidizing activity, reducing the petroleum hydrocarbon content by 80–98%.
(b) Species with medium hydrocarbon oxidizing activity, reducing the petroleum hydrocarbon content by 50–79%.
(c) Species with low hydrocarbon oxidizing activity, reducing the petroleum hydrocarbon content by 49% or less.

The group of species with low hydrocarbon oxidizing activity, represented by 43 species, was the most diverse in terms of species composition. The fungi had a weak ability to grow on petroleum hydrocarbons and were not of particular interest for further study. The group of species with medium hydrocarbon oxidizing activity was represented by 20 species of microfungi.

The group of species with high hydrocarbon oxidizing activity includes 18 strains of microfungi: *Alternaria alternata, Fusarium oxysporum, F. solani, Penicillium* canescens st. 1, st. 2, st. 3, *P. commune*, *P. decumbens*, *P. implicatum*, *P. jensenii* st. 1, *P. miczynskii* st. 1, *P. nigricans*, *P. ochrochloron*, *P. restrictum*, *P. simplicissimum* st. 1, st. 2, *P. velutinum*, and *Ulocladium consortiale*. Among these, in terms of species diversity, the genus Penicillium prevailed, the representatives of which are also resistant to other types of pollution [42,43].

The most intensive petroleum hydrocarbons decomposed five species of microfungi: *Penicillium canescens* st. 1, *P. simplicissimum* st. 1, *P. commune, P. ochrochloron,* and *P. restrictum*. One day after the start of the experiment, 6 to 18% of hydrocarbons decomposed. This grew in 3 days to 16 to 49%, in 7 days to 40 to 73%, and in 10 days to 71 to 87%. The *Penicillium commune* exhibited the greatest degrading activity throughout the experiment.

In the experiment with associations of microorganisms, on the third day, there was a decrease in petroleum hydrocarbon content of 11 to 38%. On the seventh day, it was 17 to 83%, and on the tenth day, it was up to 94%. The associations of microfungi with bacteria were the most effective. It showed the best result on the third day of the experiment. The association of bacteria in this experiment was more passive and decreased only 20% of the petroleum hydrocarbon in 10 days. The most active association of microorganisms was used in a field experiment to assess the effectiveness of the methods for the bioremediation of contaminated soils.

### 3.2. Analysis of the Bioremediation Effectiveness in a Field Experiment

#### 3.2.1. Content of Total Petroleum Hydrocarbons

The initial content of TPH in the contaminated soil at different sites ranged from 9385 to 46,143 mg/kg (Table 3). 

**Figure 3:**
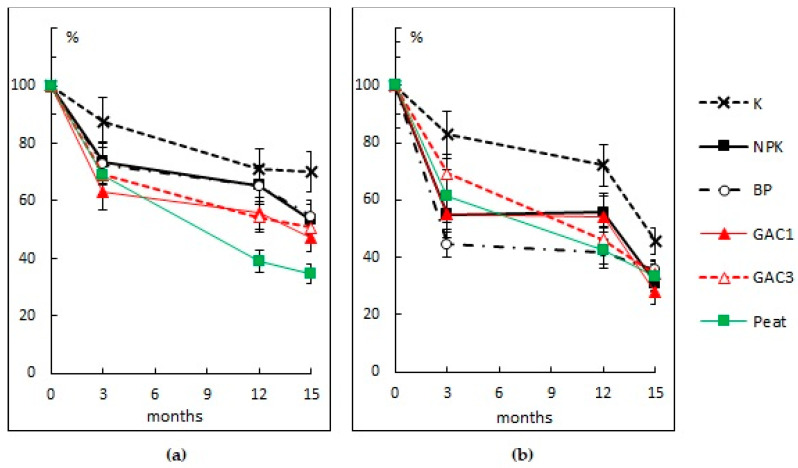
The dynamic of the TPH content (**a**) and high-molecular polar organic compounds (**b**) in the contaminated soil with different methods of bioremediation.

The TPH content in the soil in variant A decreased by 30% over 15 months due to the optimization of the air regime as a result of the periodic mixing of the upper layer. The application of mineral fertilizers decreased the concentration of hydrocarbons in the contaminated soil by an average of 47%. The use of the HOM association did not give additional advantages and reduced the TPH content by 45% from the initial value. The efficiency of the sorption-biological method of purification with the addition of GAC was 49–53%. The maximum efficiency was observed when we used peat: the TPH content decreased by an average of 65% (Figure 3a).

The content of extractable polar organic compounds (not hydrocarbons) decreases by 65–72% when using various methods of bioremediation and by 54% without bioremediation (Figure 3b).

The minimum rate of hydrocarbon decay was at the variants K and BP when we used the HOM association: 24 and 28 mg TPH/kg per day, respectively. With the sorption-biological method of purification and using GAC, the rate of hydrocarbons decay was 42–45, and with the addition of peat, it was 38 mg TPH/kg per day. The average rate of TPH decay when using the biostimulation method was 47 mg TPH/kg per day. The maximum rate of TPH decay in all variants of the field experiment was observed in the first 3 months and reached 97–105 mg TPH/kg per day when using GAC.

The content of high-molecular polar organic compounds also decreased most intensively in the first 3 months. In both cases, in variants NPK, BP, and GAC1, we observed a decrease in the rate of decay in the winter (0–9 mg/kg per day), whereas in the variants GAC3 and Peat, the rate of decay reached 25 mg/kg per day.

The sorption-biological method was more effective than the other methods according to the results of the first growing season. An undoubted advantage of this method is the ability of the microorganisms on the sorbent to oxidize the hydrocarbons and organic compounds of other classes even in winter. Therefore, by the beginning of the second growing season, the content of the hydrocarbons in these areas decreased more.

The data from the analysis of the hydrocarbon content in the soil were used to calculate the time of their almost complete decomposition, i.e., by 99% (T99), according to the exponential dependence [44].

The studies showed that the decomposition of hydrocarbons was accelerated by 1.7 and 2.9 times with bioremediation (Table 4). When using peat, almost complete soil cleaning will occur in 5.4 years, without bioremediation—in 16.1 years.

#### 3.2.2. Soil Humidity and Temperature

**Figure 4:**
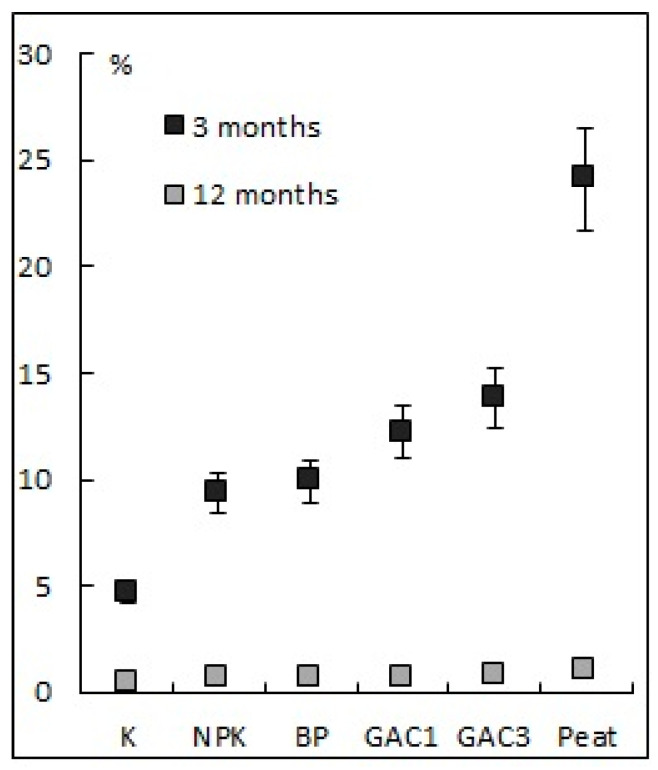
The change of the soil humidity due to the bioremediation.

The bioremediation did not significantly affect the soil temperature measured at the time of sampling in June and September. However, the temperature measured a year after the start of the study showed that on the plots with the peat, there was a tendency to decrease it. The bioremediation had a positive effect on soil humidity. The increase in soil humidity could be facilitated by the biodegradation of hydrocarbons, which could lead to the formation of water. The maximum moisture content was typical for the variant with peat. An increase in humidity can explain the observed tendency toward a decrease in temperature in variants with peat. In the autumn, the increase in soil humidity on the reclaimed sites was more pronounced than in the summer, when the moisture content dropped to a minimum (Figure 4).

#### 3.2.3. Actual Acidity

The acidity of the soil is one of the most important indicators of its condition and affects the vital activity of soil microorganisms as well as the speed and direction of the chemical and biochemical processes. Soil pH influences biodegradation through its effect on microbial activity, microbial community and diversity, enzymes that aid in the degradation processes. The optimum pH for biodegradation is alkaline or slightly acid [45]. pH values between 6.5 and 8.0 are considered optimum for oil degradation [46]. 

**Figure 5:**
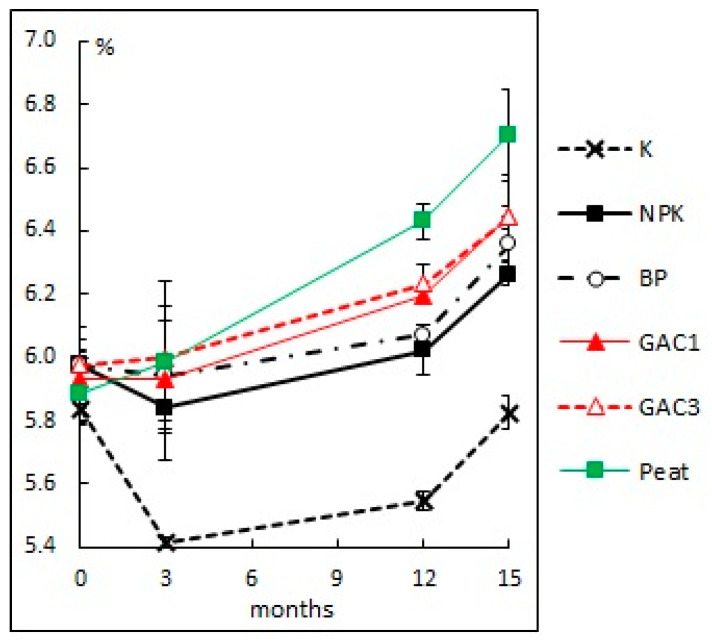
The change of soil pH with various methods of bioremediation.

Liming, the use of GAC and peat in the process of cleaning contaminated areas, increased the soil pH by 0.3–0.8 units (Figure 5). At the same time, when the upper layer of the contaminated soil is only loosened, soil acidification is observed, which can adversely affect the state of the soil microbial community and is unfavorable for plants.

The change of soil pH is not only a consequence of liming but also the buffering effect of some elements (potassium, calcium, magnesium cations, etc.) contained in the activated carbons, which was previously observed in laboratory experiments [29,30,47]. Thus, the use of GAC reduced the negative consequences of applying high doses of mineral fertilizers during bioremediation and maintained the soil pH in the optimal range for most plants and microorganisms.

#### 3.2.4. The Number of Hydrocarbon-Oxidizing Microorganisms

**Figure 6:**
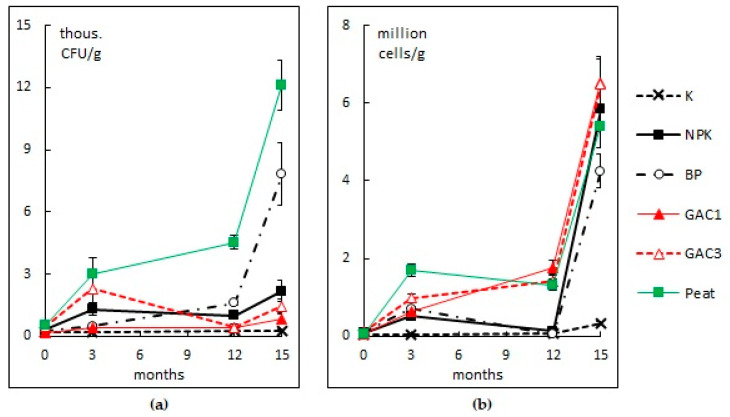
The dynamic of the number of hydrocarbon-oxidizing microfungi **(a)** and bacteria **(b)** in soil with different methods of bioremediation.

The number of hydrocarbon-oxidizing microfungi in the soil varied from 0.15 to 0.52 thousand. CFU/g (Figure 6a). As a result of biostimulation and sorption-biological treatment of the site, an increase in the number of microfungi was already noted (variants NPK, GAC3, and Peat) in the first months. After 15 months, the maximum number of microfungi was observed in variant BP, which is due to the repeated introduction of the association of HOM and variant Peat (7.82 and 12.13 thousand CFU/g, respectively). The high number of microfungi with variant Peat is due to the microbial pool of the peat itself, which, according to the results obtained, was more effective than the microbial pool of the HOM association. In variant K, the number of microfungi did not change during the experiment. This may be the result of a less favorable habitat for microfungi (low organic matter content, very low humidity in the summer, and the high temperature and pH values of the substrate). Further, it was noted that the number of microfungi increases in the autumn under more favorable living conditions.

Observations of the bacterial component of soil samples during the second growing season showed that the maximum number of bacteria in the soil of the recultivated areas was 6.5 million cells/g. Noteworthy is the low number of bacteria in variants NPK and BP at the beginning of the second growing season (Figure 6b), which is probably due to the very low moisture content of these samples. An increase in the bacteria number in the variants GAC1, GAC3, and Peat is associated with an improvement of the physicochemical soil properties and the appearance of an additional source of organic carbon. Throughout the growing season, no significant differences were found related to the number of bacteria in the soil and the addition of GAC and peat.

In all the samples of contaminated soils, a very low species diversity of bacteria was noted. Bacteria of the genus Pseudomonas dominated; pigmented forms of bacteria more resistant to pollution were presented. Actinomycetes were found in small numbers. The considered bioremediation methods of the petroleum-contaminated area had a positive effect on the bacteria number. Sorption-biological methods of bioremediation make it possible to increase the resistance of bacterial community to unfavorable factors, which is confirmed by a higher bacteria number at the beginning of the second growing season.

#### 3.2.5. Soil Dehydrogenase Activity

Microorganisms are the main source of enzymes entering the soil. Changes in their composition and abundance in contaminated soil also affect the activity of soil enzymes. Due to extracellular enzymatic activity, the soil metabolism can remain unchanged even in unfavorable conditions for the vital activity of microorganisms [48]. The most important and widespread oil destructor enzymes of soil microorganisms are catalase and dehydrogenase. Dehydrogenase is an intracellular enzyme, and therefore, its activity reflects the functional state of the soil microbial community [49,50].

**Figure 7:**
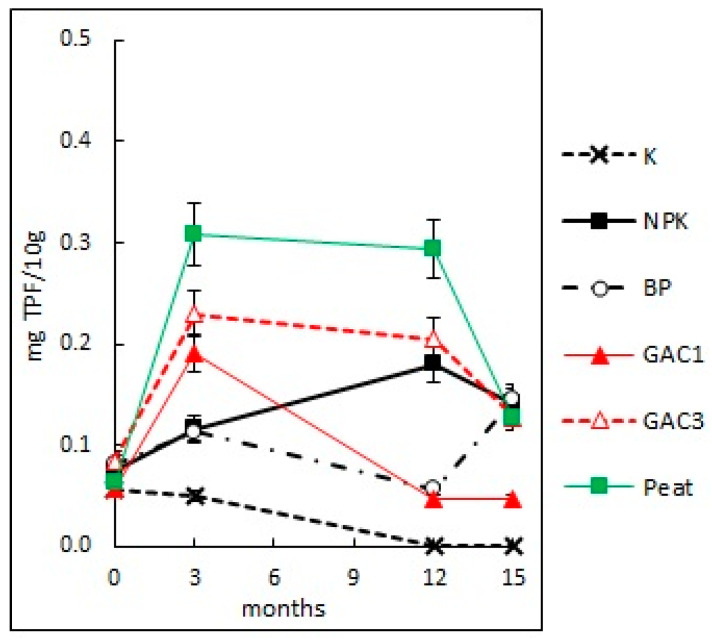
The change of soil dehydrogenase activity with various methods of bioremediation.

The dehydrogenase activity in the contaminated soil before the bioremediation was 0.06 mg TPF/10 g, which corresponds to very weak biological activity. This may indicate the inhibition of the enzymatic dehydrogenation of hydrocarbons. The maximum values of the enzyme activity were observed at the end of the first and second growing seasons in the variants NPK (0.18 mg TPF/10 g), GAC3 (0.21 mg TPF/10 g), and Peat (0.29 mg TPF/10 g; Figure 7), which, however, corresponds to weak enzymatic activity.

More high values of enzymatic activity at the beginning of the second growing season in variants GAC3 and Peat correlate with the rate of the degradation of the hydrocarbons in these variants in winter. By the end of the second growing season, the enzyme activity in all the variants, except for the variant BP, slightly decreased, despite the increase in the number of microorganisms.

## 4. Discussion

The results of the correlation analysis showed a negative correlation between the number of HOM and the content of TPH (r = 0.613–0.725 for microfungi and r = 0.626–0.730 for bacteria) and a positive correlation between the number of HOM and soil pH (r = 0.632–0.988 for microfungi and r = 0.545–0.951 for bacteria). At the same time, no correlation was found between the number of microorganisms and dehydrogenase activity, with the exception of the variant BP when using HOM association, where a positive correlation was noted (r = 0.714–0.944).

At this stage of bioremediation, the biological system is unstable. Biochemical processes in the contaminated substrate have low efficiency and multi-directionality. In addition, the environmental conditions have a great influence on the number of HOM. The research results show that, along with pH, moisture has a strong effect on biochemical processes in contaminated soils [51,52]. The activity of microorganisms decreases with low humidity, despite the temperature being close to the optimum, which is characteristic for soils with light granulometric composition and low organic content.

A number of studies demonstrate that both biostimulation and bioaugmentation increase the efficiency of soil cleansing processes from oil products. The choice of the appropriate method depends on the environmental conditions [53].

In our research the improvement of the water and air conditions (only loosening the top layer of soil) in the process of cleaning the contaminated areas was quite effective. The decrease in the TPH content in the soil was 30% over the entire observation period. The number of HOM and the activity of soil dehydrogenase did not change significantly, remaining at a very low level, which indicates the predominantly physical and chemical degradation of the hydrocarbons.

Biostimulation (i.e., using the mineral fertilizer together with dolomite flour) increased the rate of hydrocarbons decay: the TPH content in the soil decreased by an average of 47% in 15 months. There was an increase in the number of hydrocarbon-oxidizing microfungi by 6–7 times, bacteria by 17 times, and the activity of soil dehydrogenase by 2–6 times, which indicates of biochemical oxidation of hydrocarbons.

Bioaugmentation (use the HOM association) reduced the TPH content in the soil by 45% in 15 months, which is comparable to the results obtained with biostimulation. A significant increase in the number of microorganisms occurs only after the repeated introduction of the microorganism associations after 1 year.

The sorption-biological cleaning of the contaminated sites using the GAC also intensified the biochemical oxidation of hydrocarbons (we noted an increase the number of HOM and the activity of dehydrogenase). It was possible to reduce the TPH content by 47–53%. However, the effectiveness of this method does not exceed the effectiveness of biostimulation but does increase the cost of cleaning. The positive effect of the introduction of sorbents into highly contaminated soils and grounds is due to the reversible sorption of toxic hydrocarbons and their metabolites. In biodegradation of n-alkanes, a primary alcohol is usually formed, followed by an aldehyde and a monocarboxylic acid. Further degradation of the carboxylic acid proceeds by oxidation with the formation shorter fatty acids, some of which are toxic and accumulate during hydrocarbon biodegradation. Isoprenoid alkanes oxidate with the formation of dicarboxylic acids as the major degradative pathway. Cycloalkanes can be substrates for co-oxidation to form a ketone or alcohol. The bioremediation of aromatics involves the formation of a diol (followed by cleavage to a diacid) or a transdiol [54,55,56].

The sorbed hydrocarbons remain available to microorganisms. This is evidenced by an increase in the number of HOM, which occurs simultaneously with a decrease in TPH. A decrease in the toxic effect when using GAC was shown earlier in laboratory experiments [29]. The best results were obtained with the sorption-biological treatment of the peat. The content of hydrocarbons at the sites decreased by an average of 65%, which is 2.5 times more effective than in the variant A. In this variant, the maximum values of the HOM number and the activity of soil dehydrogenase were also noted. These patterns, noted by other researchers, are associated with the fact that sorbents based on GAC [57,58] and peat [59,60] provide both the sorption of petroleum hydrocarbons and are carriers of hydrocarbon-oxidizing microorganisms. However, peat may also contain various species of naturally occurring community that are capable of TPH degradation [61,62].

## 5. Conclusions

Methods of biostimulation and sorption-biological treatment can be used for the bioremediation of contaminated sites in the subarctic zone of Russia, despite the difficult climatic conditions of the locations of the contaminated areas on the slopes of mountains and a high degree of soil degradation.

Based on the results of the laboratory experiments, a scale of degrading activity of microfungi to petroleum hydrocarbons, was developed, which included three groups (with high, medium and low hydrocarbon oxidizing activity). Five species of microfungi that most intensively degraded petroleum hydrocarbons have been identified (*Penicillium canescens st. 1, P. simplicissimum st. 1, P. commune, P. ochrochloron, P. restrictum*).

For soils and ground of light granulometric composition with a low content of organic matter but with a sufficiently high temperature regime, sorption-biological treatment using peat or GAC can be a more effective method of bioremediation. With this approach, the sorbent will not only bind hydrocarbons and their toxic metabolites but will also serve as a carrier for HOM and prevent the leaching of nutrients from the soil. The introduction of sorbents (mostly peat) increases the moisture capacity of the treated soil, which leads to an increase in the activity of HOM and the efficiency of bioremediation without the need for the periodic watering of the site.

Good results were obtained due to the biostimulation of aboriginal hydrocarbon-oxidizing microbiota by the introduction of mineral fertilizers and liming. An increase in the number of microorganisms and dehydrogenase activity indicates the presence of a certain microbial potential in the soil and the possibility for the biochemical oxidation of hydrocarbons. The use of the HOM association, created based on bacteria and microfungi, which are active destructors of petroleum products, was less effective under these conditions and could be replaced by biostimulation techniques. Biostimulation techniques will reduce the cost of the treatment of the territory. The use of the considered bioremediation techniques contributes to the improvement of the ecological state of the contaminated area and to gradually recovering its biodiversity. In the future, we plan to undertake longer observations of the model areas, as well as to evaluate the effectiveness of bioremediation methods (including the various carbon sorbents) and develop recommendations for the biological purification of contaminated soils and coastal substrates from oil products in the Arctic and Subarctic.

## Figures and Tables

**Table 1:** The experiment design.

Variant	Activated Carbon (GAC), g/m^2^	N_16_P_16_K_16_, (g N, P_2_O_5_, K_2_O/m^2^)	Dolomite Flour, g CaCO_3_/m^2^	HOM Association Suspension, L/m^2^	Peat, L/m^2^
K	-	-	-	-	-
NPK	-	25	200	-	-
BP	-	25	200	2	-
GAC1	1300	25	200	-	-
GAC3	3900	25	200	-	-
Peat	-	25	200	-	16

**Table 2:** Hydrocarbon oxidizing activity of fungi species for 14 days (the initial concentration of petroleum products is 1% by volume in the medium).

Species of Microfungi	Petroleum Degradation, % of the Initial Amount	Degradation Activity, g Petroleum /g Microfungi	Dry Biomass, g
*group I—high activity*			
*Penicillium commune* Thom	98	0.414	0.596
*P. canescens* (st.1) Sopp	98	0.372	0.867
*P. simplicissimum* (st.1) (Oudem.) Thom	96	0.353	0.687
*P. canescens* (st.2) Sopp	95	0.335	0.758
*P. restrictum* J.C. Gilman & E.V. Abbott	95	0.330	0.738
*P. ochrochloron* Biourge	94	0.337	0.615
*P. velutinum* J.F.H. Beyma	93	0.253	0.335
*Ulocladium consortiale* (Thum.)E.G.Simmons	92	0.343	0.518
*P. implicatum* Biourge	92	0.327	0.530
*P. decumbens* Thom	92	0.266	0.810
*P. canescens* (st.3) Sopp	88	0.229	0.651
*P. simplicissimum* (st.2) (Oudem.) Thom	87	0.311	0.519
*P. miczynskii* (st.1) K.M. Zaleski	85	0.294	0.573
*P. spinulosum* Thom.	84	0.245	0.847
*P. jensenii* (st.1) K.M. Zaleski	84	0.312	0.667
*Fusarium solani* (Mart.) Sacc.	84	0.310	0.251
*Alternaria alternata* (Fr.) Keissl.	83	0.304	0.515
*F. oxysporum* (st.1) Schltdl.	80	0.311	0.268
*group II—medium activity*			
*P. aurantiogriseum* (st.1) Dierckx	79	0.269	0.620
*Rhizopus stolonifer* (Ehrenb.) Vuill.	76	0.285	0.485
*P. adametzii* K.M. Zaleski	75	0.267	0.716
*P. glabrum* (Wehmer) Westling	74	0.248	0.406
*P. spinulosum* (st.2) Thom	74	0.281	0.463
*P. miczynskii* (st.2) K.M. Zaleski	73	0.265	0.525
*Lecanicillium lecanii* (Zimm.) Zare & W. Gams	72	0.298	0.352
*Stachybotrys echinata* (Rivolta) G. Sm.,	70	0.239	0.406
*P. canescens* (st.4) Sopp.	70	0.295	0.466
*Trichoderma viride* Pers.	67	0.233	0.283
*P. aurantiogriseum* (st.2) Dierckx	67	0.247	0.418
*P. nalgiovense* Laxa	66	0.266	0.628
*Umbelopsis isabellina* (Oudem.) W. Gams	63	0.294	0.415
*Cephalotrichum stemonitis* (Pers.) Nees	62	0.257	0.563
*Talaromyces stipitatus* C.R. Benj.	62	0.270	0.361
*Chaetomium bostrychodes* Zopf	60	0.294	0.365
*Acremonium egyptiacum* (J.F.H. Beyma) W. Gams,	57	0.215	0.391
*Fusicolla merismoides* (Corda) Gräfenhan, Seifert & Schroers	57	0.218	0.224
*Wallrothiella subiculosa* Höhn.	55	0.241	0.300
*P. multicolor* Grig.-Man. & Porad.	50	0.231	0.407
*group III—low activity*			
*L. psalliotae* (Treschew) Zare & W. Gams	46	0.233	0.369
*Clonostachys rosea* (Link) Schroers, Samuels, Seifert & W. Gams	44	0.281	0.402
*Umbelopsis longicollis* (Dixon-Stew.) Y.N Wang, X.Y. Liu & R.Y. Zheng	41	0.230	0.391
*Cephalosporium bonordenii* Sacc.	37	0.204	0.191
*Pseudogymnoascus pannorum* (Link) Minnis & D.L. Lindner	33	0.233	0.185
*Scopulariopsis communis* (st.1) Bainier	33	0.195	0.168
*P.thomii* Maire	31	0.196	0.444
*Aspergillus fumigatus* Fresen.	30	0.287	0.438
*P.jensenii* K.M. Zaleski	30	0.241	0.332
*Tr. koningii* Oudem.	29	0.162	0.116
*P.melinii* Thom	29	0.274	0.419
*P. aurantiogriseum* (st.3) Dierckx	28	0.245	0.461
*Phoma eupyrena* Sacc.	28	0.236	0.513
*Torula herbarum* (Pers.) Link	27	0.180	0.186
*Mucor hiemalis* Wehmer	25	0.149	0.104
*Ph. herbarum* Westend.	25	0.205	0.236
*Acr. charticola* (Lindau) W. Gams	24	0.213	0.234
*Tr. polysporum* (Link) Rifai	23	0.136	0.044
*Amorphotheca resinae* Parbery	20	0.170	0.174
*Gibberella fujikuroi* (Sawada) Wollenw.	20	0.170	0.293
*Acr. rutilum* W. Gams	20	0.117	0.123
*Tr. aureoviride* Rifai	19	0.162	0.165
*Clad. cladosporioides* (Fresen.) G.A. de Vries	19	0.160	0.071
*Gibellulopsis nigrescens* (Pethybr.) Zare, W. Gams & Summerb.	17	0.222	0.183
*P.aurantiogriseum*(st.4) Dierckx	17	0.122	0.327
*Aureobasidium microstictum* (Bubák) W.B. Cooke	17	0.110	0.095
*Sc. communis* (st.2) Bainier	15	0.109	0.165
*Gongronella butleri* (Lendn.) Peyronel & Dal Vesco	13	0.107	0.109
*Ph. glomerata* (Corda) Wollenw. & Hochapfel	13	0.029	0.279
*M.circinelloides* Tiegh.	10	0.116	0.163
*Rodotorula sp.*	10	0.087	0.128
*Aur. pullulans* (de Bary & Löwenthal) G. Arnaud	9	0.054	0.097
*Cph. nanum* (Ehrenb.)S.Hughes	9	0.050	0.090
*P. corylophilum* Dierckx	9	0.091	0.501
*Streptothrix luteola* Foul. & P.C. Jones,	8	0.107	0.311
*Tl. flavus* (Klocker) Stolk et Samson	8	0.126	0.289
*P. raistrickii* G. Sm.	7	0.084	0.423
*Asp. repens* (Corda) Sacc.	6	0.074	0.100
*T. allii* (Harz) Sacc.	5	0.083	0.141
*Botrytis cinerea* Pers.	5	0.065	0.164
*F. oxisporum* Schltdl.	4	0.053	0.178
*P. spinulosum* Thom	4	0.033	0.119
*Humicola grisea* Traaen	3	0.020	0.280

**Table 3:** The initial content of TPH and organic matter, humidity, pH and temperature of soil in the contaminated area.

TPH, mg/kg	Organic Matter, %	pH	Humidity, %	Temperature, °C
26,548 ± 2299	5.04 ± 0.29	5.93 ± 0.03	10.2 ± 3.1	21.5 ± 1.1

**Table 4:** Time of practically full degradation (Т99) and degradation to approximate permissible concentration (Т_APC_, 700 mg/kg) of petroleum hydrocarbons in soil.

Variant	The Rate Constant of Hydrocarbons Decomposition	T_APC_ (Days)	T99 (Days)
K	0.00079	4892	5816
NPK	0.00140	2699	3298
BP	0.00135	2428	3420
GAC1	0.00168	2104	2746
GAC3	0.00151	2501	3053
Peat	0.00235	1479	1960
